# Commentary: Add a ventricular assist device? Add a stent? A tree of decisions for small univentricular hearts

**DOI:** 10.1016/j.xjtc.2021.10.007

**Published:** 2021-10-09

**Authors:** Douglas M. Overbey, Nicholas Andersen, Joseph W. Turek

**Affiliations:** a Department of Surgery, Duke University Medical Center, Durham, NC; b Division of Cardiovascular and Thoracic Surgery, Duke University Medical Center, Durham, NC; c Duke Children's Pediatric & Congenital Heart Center, Duke Congenital Heart Research & Training Laboratory, Duke University, Durham, NC

**Figure:**
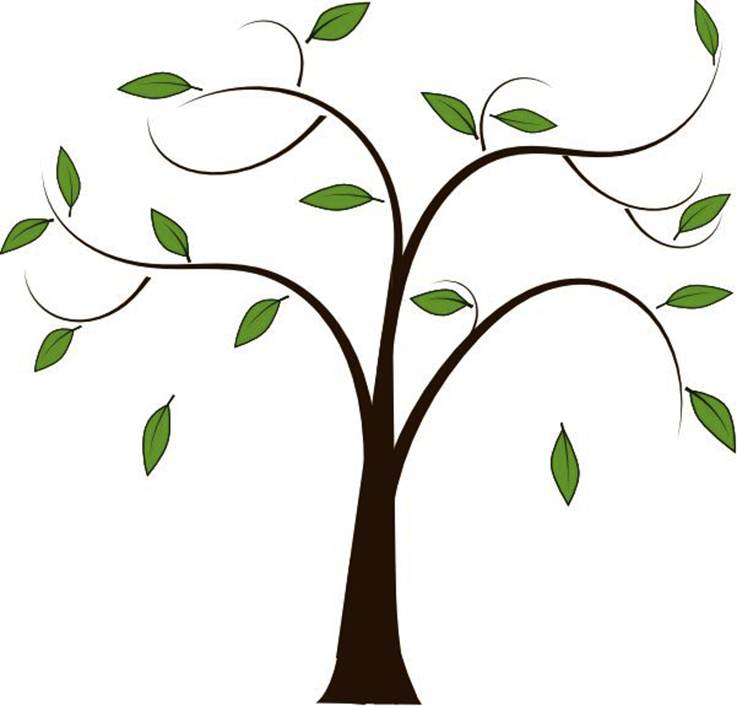
A decision tree for supporting neonates and infants with single-ventricle physiology.

**Central Message**

HLHS and HRHS hybrid VAD cannulation techniques are described for high-risk neonates and infants.

See Article page 194.

Bleiweis and colleagues^1^ present a useful decision tree for neonates and infants with single-ventricle circulation based on right- or left-sided malformation, noncardiac risk factors, and major cardiac risk factors. This comprehensive strategy includes thoughtful descriptions of conventional neonatal palliation, hybrid approaches, and mechanical support. The authors capture the heterogeneity of single-ventricle heart disease and describe selection criteria, management strategies, and pitfalls for using ventricular assist device (VAD) support as a bridge to transplantation. Detailed pictures and technical details are provided for each of the 3 palliation strategies.

The techniques of VAD implantation are categorized based on the need for pulmonary blood flow and are similar to other reports.^2^^,^^3^ For hypoplastic left heart syndrome, the authors prefer cannulation of the atrium and main pulmonary artery, along with bilateral pulmonary artery bands, ductal stent, and atrial septectomy, if needed. For hypoplastic right heart syndrome, the authors describe cannulation of the atrium and aorta, along with ductal stent and atrial septectomy, if needed. If a shunt is needed for hypoplastic right heart syndrome rather than a ductal stent, the authors recommend placement of a graft from the aortic chimney graft versus directly originating from the aorta. The hybrid components involve ductal stent placement either in the operating room with fluoroscopy, or intraoperative transfer to the cardiac catheterization laboratory.

Logistics and management details include a multidisciplinary team, VAD management parameters, and anticoagulation strategies. Single-center outcomes are described with 15 patients who required a VAD with palliation. These patients had unfavorable coronary anatomy or ischemia, heart failure, or cardiogenic shock necessitating this approach. The main complication was stroke (22%-33%). Overall survival for the 15 neonates and infants was 53.3%, which must be considered in the context of waitlist and age group.^4^

The strength of this study is the comprehensive categorizations as an expert technical review. The indications of each strategy and details provided regarding each specific approach are practical and transferrable. Limitations include a small number of patients and widely variable anatomy and physiology in this population.

Overall, this analysis puts context to univentricular mechanical support as a bridge to transplantation and describes an excellent algorithm to select and palliate neonates appropriate for each strategy. The surgical techniques are described in detail and should have broad applicability. The advantages of stabilizing with a VAD include extubation, enteral nourishment, and transplant waitlist optimization. High-risk palliative strategies, primary transplantation waitlist, and VAD outcomes should all be weighed at the institutional level to fine-tune the decisions, but hybrid VAD should be a part of the congenital surgeon's armamentarium in approaching the single-ventricle neonate to maximize survival while awaiting transplantation.^1^

## References

[1] Bleiweis M., Fudge J.S., Peek G.J., Vyas H.V., Cruz Beltran S., Pitkin A.D. (2022). Ventricular assist device support in neonates and infants with a failing univentricular circulation. J Thorac Cardiovasc Surg Tech.

[2] Miller J.R., Lancaster T.S., Callahan C., Abarbanell A.M., Eghtesady P. (2018). An overview of mechanical circulatory support in single-ventricle patients. Transl Pediatr.

[3] Andersen N.D., Kirmani S., Turek J.W. (2019). Commentary: shunted single-ventricle neonatal ventricular-assist device support: are we nearing a consensus strategy?. J Thorac Cardiovasc Surg.

[4] Peng D.M., Koehl D.A., Cantor R.S., McMillan K.N., Barnes A.P., McConnell P.I. (2019). Outcomes of children with congenital heart disease implanted with ventricular assist devices: an analysis of the Pediatric Interagency Registry for Mechanical Circulatory Support (Pedimacs) [Erratum in: *J Heart Lung Transplant*. 2020;39:1512-4.]. J Heart Lung Transplant.

